# Supplementary material for: Targeted next-generation sequencing of bronchoalveolar lavage fluid for pathogen detection in connective tissue disease-related pulmonary infections
Source: Microbiol Spectr. 2025 Nov 18;14(1):e02550-25. doi: 10.1128/spectrum.02550-25 (PMC12772244; doi:10.1128/spectrum.02550-25)
Supplement: Supplemental tables — Tables S1 to S3. [file spectrum.02550-25-s0001.docx]

**Table S1.** CTD cases included in the study

| Disease | Case | | Percentage (%) | |
| --- | --- | --- | --- | --- |
| RA | | 26 | | 20.97 |
| SLE | | 25 | | 20.16 |
| DM | | 17 | | 13.71 |
| pSS | | 13 | | 10.48 |
| AAV | | 11 | | 8.87 |
| PM | | 10 | | 8.06 |
| ASS | | 6 | | 4.84 |
| UCTD | | 5 | | 4.03 |
| SD | | 4 | | 3.23 |
| SSc | | 4 | | 3.23 |
| BD | | 2 | | 1.61 |
| OS | | 1 | | 0.81 |

**Table S2.** Pathogens Detected by CMTs

| Disease | Case | Percentage (%) |
| --- | --- | --- |
| **Bacteria** | 23 | 41.82 |
| *K. pneumoniae* | 8 | 14.55 |
| *P. aeruginosa* | 7 | 12.73 |
| *E. faecium* | 3 | 5.45 |
| *E. coli* | 2 | 3.64 |
| *H. influenzae* | 1 | 1.82 |
| *S. aureus* | 1 | 1.82 |
| *A. baumannii* | 1 | 1.82 |
| **Viruses** | 13 | 23.64 |
| EBV | 6 | 10.91 |
| Adv | 3 | 5.45 |
| CMV | 2 | 3.64 |
| Flu B | 1 | 1.82 |
| RSV | 1 | 1.82 |
| **Fungi** | 12 | 21.82 |
| *C. albicans* | 6 | 10.91 |
| *A. fumigatus* | 2 | 3.64 |
| *C. famata* | 2 | 3.64 |
| *C. glabrata* | 2 | 3.64 |
| **M. pneumoniae** | 7 | 12.73 |

**Table S3.** Pathogens Detected by tNGS in Bronchoalveolar Lavage Fluid

| Pathogen | Case | Percentage (%) |
| --- | --- | --- |
| **Bacteria** | 70 | 50.72 |
| *H. influenzae* | 14 | 10.14 |
| *P. jirovecii* | 13 | 9.42 |
| *K. pneumoniae* | 9 | 6.52 |
| *P. aeruginosa* | 8 | 5.80 |
| *S. aureus* | 7 | 5.07 |
| *M. tuberculosis* | 5 | 3.62 |
| *M. catarrhalis* | 4 | 2.90 |
| *S. pneumoniae* | 2 | 1.45 |
| *E. coli* | 2 | 1.45 |
| *S. intermedius* | 1 | 0.72 |
| *N. cyriacigeorgica* | 1 | 0.72 |
| *T. whipplei* | 1 | 0.72 |
| *S. mitis group* | 1 | 0.72 |
| *E. faecalis* | 1 | 0.72 |
| *N. farcinica* | 1 | 0.75 |
| **Viruses** | 37 | 26.81 |
| CMV | 8 | 5.80 |
| SARS-CoV-2 | 6 | 4.35 |
| EBV | 5 | 3.62 |
| *Influenza A virus* | 3 | 2.17 |
| HSV-1 | 3 | 2.17 |
| HHV-5 | 2 | 1.45 |
| HAdV-3 | 1 | 0.72 |
| *Parvovirus B19* | 1 | 0.72 |
| hRSV-B | 1 | 0.72 |
| hRSV-A | 1 | 0.72 |
| *Human Coronavirus）* | 1 | 0.72 |
| hPIV-3 | 1 | 0.72 |
| hPIV-1 | 1 | 0.72 |
| *Influenza C virus* | 1 | 0.72 |
| RV-B | 1 | 0.72 |
| RV-A | 1 | 0.72 |
| **Fungi** | 26 | 18.84 |
| *C. albicans* | 12 | 8.70 |
| *A. fumigatus* | 5 | 3.62 |
| *A. baumannii* | 4 | 2.90 |
| *C. glabrata* | 2 | 1.45 |
| *C. neoformans* | 1 | 0.72 |
| *T. marneffei* | 1 | 0.72 |
| *A. flavus* | 1 | 0.72 |
| ***M. pneumoniae*** | 5 | 3.62 |
